# Supplementary material for: Extracellular Vesicles as Markers of Liver Function: Optimized Workflow for Biomarker Identification in Liver Disease
Source: Int J Mol Sci. 2023 Jun 1;24(11):9631. doi: 10.3390/ijms24119631 (PMC10253831; doi:10.3390/ijms24119631)
Supplement: Supplementary file 1 [file ijms-24-09631-s001.zip › Supplementary.File_S1.pdf]

Separation and isolation of EVs from serum, plasma and TC medium with PEG.

24% PEG-1.5M NaCl solution preparation _

Dissolve in **24gr of PEG 8000 in 90 mL** ddH2O by heating (60 to 70°C) and mixing (30 to 60 minutes). Once PEG is dissolved add **8.7 gr of NaCl to the PEG solution**. Adjust volume **to 100 mL with ddH2O** and filter sterile with **0.45 µM filters**. Store stocks frozen (-20C). Keep a working aliquot at 4°C for no more than 30 days.

Isolation of EVs from serum or plasma _

Centrifuge Plasma/Serum 10 minutes 10000 rcf/4°C to remove larger debris. Transfer serum in a fresh Eppendorf tubes.

Optional: If required, label vesicles by addition of 1 µl of SytoRNA Select/Cell Mask for 1 ml of serum. Vortex and incubate 15 minutes at RT before proceeding with the nest step

Add PEG-NaCl (see below) with a ratio of 5:1 (**i.e., 100 µL plasma+20 µL PEG-NaCl**). Vortex and incubate 30 in ice. After incubation spin for 10 min/10000 rcf/4°C. This precipitation step will remove 99% of plasma/serum proteins. For higher purity dissolve pellets in PBS and repeat isolation.

NOTE: Final concentrations AFTER addition to plasma/serum are: 4% PEG, 250 mM NaCl.

At the end of the spin, EVs are enriched in the pellets. If negative control required collect supernatant in a new epi-tube (EVs depleted sera) and store -20°C.

Optional: To remove EVs surface proteins, dissolve pellets in PBS and incubate samples ON/37°C with **200 µg/ml** of PTK, then isolate EVs according to standard protocol. [e.g., PTK sln. (ROTH, stock, 10 mg/ml)].

If vesicles are to be used in further analysis go to (**A**), for RNA extraction go to (**B**).

(**A**): Add on the pellets 1 ml of ice cold PBS (**DO NOT DISSOLVE!**), spin 1 min/10000 rcf/4°C, Repeat previous point. To completely remove PBS spin 1 min/10000 rcf/4°C completely remove the water. Either freeze the pellets or add 100 – 200 µl of cold PBS to the pellets. Incubate 10 – 15 minutes and dissolve pellets by pipetting.

(**B**): Rinse pellets as in point A, dissolve pellets in PBS and add 500 ul Qiazol to EVs pellets, vortex and incubate for 3 minutes RT. Add 200 ul chloroform and proceed with modified miRNeasy isolation kit.

Mix for 20-30 seconds and spin for 15 minutes/max speed/4°C.

Collect aqueous phase into a fresh eppendorf tube

Add VOL/VOL of chloroform, vortex and spin for 5 minutes/max speed/4°C.

While samples are spinning prepare miRNeasy columns in the same number as samples.

Separation and isolation of EVs from serum, plasma and TC medium with PEG.

Collect aqueous phase into a fresh eppendorf tube, add 1.5 volumes of 100% ethanol and mix.

Load samples on columns (max 700 ul) and spin 30 seconds/8000 rpm/RT If volume exceed 700 ul. Repeat previous step until all the samples has been loaded on the miRNeasy columns.

Remember to discard the flow through and to keep the collection tube for the following spin

Wash 1x with 250 ul of RWT buffer and spin 30 seconds/8000 rpm/RT

Wash 2x with 400 ul RPE buffer and spin 30 seconds/8000 rpm/RT Spin 60 seconds/max speed rpm/RT Carefully discharge collection tube and place column on clean eppendorf tube.

Load in the center of the column 40 ul of elution buffer.

Incubate 1 minute/RT

Spin 30 seconds/8000 rpm/RT

Reload the throw through on column and incubate 1 minute and Spin 60 seconds/8000 rpm/RT

Eluted RNA samples can be stored at -80C until use

Isolation of EVs from Conditioned Tissue culture medium _

NOTE: to obtain enough EVs for downstream analysis we normally start from at least 50 mL of conditioned (serum free) tissue culture medium.

Centrifuge conditioned **TC medium** for 10 minutes 10000 rcf/4°C to remove larger debris.

Transfer TC medium to in a fresh falcon tube, proceed directly or freeze until use.

Add up to 15 mL of TC medium to a Vivaspin column (pre-washed with 10 mL PBS) and spin 15-20 minutes 4°C/2000 rcf. Collect TC medium containing concentrated EVs.

Repeat until 2-4 mL of concentrate TC medium are left in the column.

Add PEG-NaCl with a ratio of 1:1 (**i.e., 500µL TC medium+500 µL PEG-NaCl**).

Vortex and incubate ON at 4°C. After incubation spin for 10 min/10000 rcf/4°C.

The resulting pellet will be large and fluid. Carefully remove supernatant and dissolve pellets in 100-500 µL PBS, based on the size of the pellet.

Repeat EVs precipitation by adding PEG-NaCl with a ratio of 5:2 (**i.e., 100 µL EVS in PBS +40 µL PEG-NaCl**). Vortex and incubate 10 minutes in ice, and spin for 10 min/10000 rcf/4°C.

Dissolve pellets in 100-200 µL PBS

Separation and isolation of EVs from serum, plasma and TC medium with PEG.

Repeat EVs precipitation by adding PEG-NaCl with a ratio of 5:1 (**i.e., 100 μL EVS in PBS +20 μL PEG-NaCl**). Vortex and incubate 10 minutes in ice, and spin for 10 min/10000 rcf/4°C.

Dissolve pellets in 50-100 μL PBS and use 1 or 2 μL in NTA quantification (diluted 1:1500) and store the remaining EVs at -20°C.

Optional: To remove EVs surface proteins, dissolve pellets in PBS and incubate samples ON/37°C with **200 μg/ml** of PTK, then isolate EVs according to standard protocol. [e.g., **PTK sln. (ROTH, stock, 10 mg/ml)**].

Optional: If required, label vesicles by addition of **1 μl of SytoRNA** Select for 1 mL of serum. Vortex and incubate 15 minutes at RT before proceeding with the nest step

For proteomics, we need to remove the PEG. Take the **Pur-A-Lyzer Mini columns** for dialysis, and by following instructions dialyze the samples in PBS
